# The role of inflammatory indices for the prediction of preeclampsia in the first trimester: a case-control study from a tertiary center

**DOI:** 10.1590/1806-9282.20241231

**Published:** 2025-05-02

**Authors:** Göksun İpek, Atakan Tanaçan, Nazan Görmüşer, Zahid Ağaoğlu, Ayça Peker, Özgür Kara, Dilek Şahin

**Affiliations:** 1 Turkish Ministry of Health, Ankara City Hospital, Department of Obstetrics and Gynecology, Division of Perinatology – Ankara, Turkey.; 2 Cihanbeyli State Hospital, Department of Obstetrics and Gynecology – Konya, Turkey.; 3 University of Health Sciences, Turkish Ministry of Health, Ankara City Hospital, Department of Obstetrics and Gynecology, Division of Perinatology – Ankara, Turkey.

**Keywords:** Preeclampsia, First trimester, Inflammation

## Abstract

**OBJECTIVE:**

The aim of this study was to evaluate the role of systemic inflammation response index, systemic immune-inflammation index, platelet/hemoglobin ratio, and other defined low-grade inflammatory indices in predicting preeclampsia.

**METHODS:**

The presented retrospective case-control study was conducted on 304 patients diagnosed with preeclampsia and 240 low-risk pregnant women who gave birth between 2019 and 2021 in Ankara Bilkent City Hospital, a tertiary center. Patient information was obtained from the hospital database. Patients diagnosed with preeclampsia, along with possible predictive indices in the first trimester, were evaluated to predict the development of preeclampsia. The indices were neutrophil/lymphocyte ratio, AST/platelet ratio, platelet/lymphocyte ratio, lymphocyte/monocyte ratio, platelet/hemoglobin ratio, creatinine/platelet ratio, systemic immune-inflammation index (neutrophil×platelet/lymphocyte), and systemic inflammation response index (neutrophil×monocyte/lymphocyte). These indices were calculated from the first-trimester routine blood test results and compared between preeclampsia and control groups. The indices were also evaluated for the predictive value regarding the severity and onset time of the disease.

**RESULTS:**

In the first trimester, hemoglobin counts were lower in the preeclampsia group, whereas creatinine and monocyte counts were higher. The platelet/hemoglobin count ratio was significantly higher in the preeclampsia group, with a p-value of 0.025. According to receiver operating characteristic analyses, a platelet/hemoglobin count ratio of 21.41 was identified as the optimal cut-off value for the disease prediction.

**CONCLUSION:**

Systemic inflammation response index and platelet/hemoglobin ratio were evaluated along with the other indices to predict preeclampsia. The platelet/hemoglobin ratio was found to be higher in the preeclampsia group in the first trimester, making it a promising index for preeclampsia prediction.

## INTRODUCTION

Preeclampsia is a multisystem disorder encountered in 2–8% of pregnancies. The disease mostly occurs after mid-pregnancy, leading to maternal and fetal mortalities and morbidities^1^. Improving the outcome of preeclampsia necessitates the earliest identification of high-risk pregnancies for the most appropriate and early management. Preeclampsia was considered a disorder characterized by defective placentation and low-grade inflammation. Low-grade inflammation refers to increased proinflammatory cell counts without a sign of inflammatory disease^2^. The proinflammatory biomarkers were mentioned in preeclampsia as the causes of endothelial dysregulation and increased lymphocyte and neutrophil responses^3^.

Systemic inflammatory response markers were available from readily taken simple blood tests and were widely used to diagnose many diseases. In the literature, hemogram-derived indices have been used and considered valuable inflammation markers in various conditions such as survival in intensive care, gastrointestinal diseases, thyroiditis, bowel diseases, and COVID-19 infection^4–7^. In obstetrics, inflammatory indices were also evaluated for conditions that were thought to underline inflammatory etiology, such as preterm delivery and intrahepatic cholestasis of pregnancy^8,9^.

Preeclampsia is also considered an inflammatory origin disease. However, the published studies remained controversial for preeclampsia diagnosis and prediction, and the published results were predelivery evaluations of already occurring diseases^2,10^.

The aim of this study was to compare all possible low-grade inflammatory indices in preeclampsia and determine the most efficient predictive marker for the first trimester. There was no such comprehensive study for predicting preeclampsia in the first trimester. Using previously obtained blood parameters for prediction is inexpensive, rapid, and easily applicable in clinical practice.

## METHODS

The present retrospective case-control study was conducted on pregnant women diagnosed with preeclampsia and low-risk pregnant women as the control group. Patient information was obtained from the hospital database. Patients diagnosed with preeclampsia, who gave birth between 2019 and 2021, had a singleton pregnancy, were between 17 and 45 years of age, and had no systemic diseases other than hypertension, were included. The control group consisted of low-risk pregnant women with no chronic disease or medication use, who gave birth during the same timeline as the preeclampsia group included in the study. Mild and severe preeclampsia criteria were determined based on ACOG guidelines^11^. This study was approved by the "Institutional Review Board of the University of Health Sciences Turkey, Ankara Bilkent City Hospital Ethics Committee" (approval number: E2-22-2376).

The possible predictive indices were determined as NLR (neutrophil/lymphocyte count ratio), PLR (platelet/lymphocyte count ratio), LMR (lymphocyte/monocyte ratio), APRI (AST/platelet count ratio), platelet/hemoglobin count ratio, creatinine/platelet count ratio, **systemic immune-inflammation index** (SII) (neutrophil×platelet/lymphocyte), and **systemic inflammation response index** (SIRI) (neutrophil×monocyte /lymphocyte count). The indices were calculated from the first-trimester routine blood test results in the first antenatal visit and compared between groups. Then, the preeclampsia group was divided according to the disease severity and diagnosis time. The indices were also compared between subgroups.

The statistical analyses were carried out using USA's Statistical Package for the Social Sciences version 23. The normality analysis of parameters was done based on the Shapiro-Wilk test. Descriptive statistics were presented as the median and interquartile range (IQR) due to the inconsistency with a normal distribution. The Mann-Whitney U test was used to compare the parameters between the groups. Categorical variables were presented as numbers and percentages. The chi-square test was used to compare categorical variables between groups. A receiver operating characteristic (ROC) analysis was used to assess the predictive performance of the platelet/hemoglobin count ratio in preeclampsia development. The Youden Index was used to determine optimal cut-off values. Statistical significance was defined as a p-value of 0.05 with a 95% confidence interval.

## RESULTS

The study was conducted on 544 participants, including 304 patients as the preeclampsia group and 240 low-risk pregnant women as the control group. Maternal demographic characteristics for the preeclampsia and the control groups are shown in Table 1.

**Table 1:** Maternal demographic characteristics and laboratory findings and index comparison between preeclampsia and control groups.

Variable		Control group (n=240)	Preeclampsia group (n=304)	p
Maternal indices				
	Maternal age (years)	27 (7)	30 (10)	**0.001**
	Gravidity	2 (2)	2 (3)	0.242
	Parity	1 (1)	1 (2)	0.573
Laboratory parameters				
	Hemoglobin (g/dL)	13.0 (1.2)	12.6 (1.4)	**0.005**
	WBC (10^9^/L)	8.70 (2.8)	8.74 (2.8)	0.068
	Platelet (10^9^/L)	265.5 (68.0)	272.0 (81.0)	0.138
	AST (U/L)	17.0 (6.0)	16.9 (5.8)	0.973
	ALT (U/L)	15.0 (9.0)	15.0 (11.0)	0.954
	Creatinine (mg/dL)	0.53 (0.1)	0.56 (0.1)	**0.035**
	Neutrophil (10^9^/L)	5.98 (2.3)	6.09 (2.5)	0.169
	Lymphocyte (10^9^/L)	1.91 (0.7)	1.98 (0.8)	0.201
	Monocyte (10^9^/L)	0.44 (0.2)	0.49 (0.3)	**0.004**
Indices				
	SIRI	1.30 (1.06)	1.48 (1.04)	0.072
	SII	787.97 (535.8)	814.75 (501.0)	0.475
	APRI	0.630 (0.03)	0.605 (0.03)	0.295
	NLR	3.025 (1.73)	3.005 (1.53)	0.829
	PLR	130.51 (55.76)	135.11 (64.21)	0.820
	LMR	4.33 (2.10)	4.14 (1.93)	0.073
	Creatinine/platelet	0.002 (0)	0.002 (0)	0.692
	Platelet/hemoglobin	20.81 (5.92)	21.49 (7.84)	**0.025**

All variables were presented as medians and interquartile ranges (IQR). ALT, alanine aminotransferase; AST, aspartate aminotransferase; SIRI, systemic inflammation response index; SII, systemic immune-inflammation index; APRI, AST/platelet ratio index; NLR, neutrophil/lymphocyte ratio; PLR, platelet/lymphocyte ratio; LMR, lymphocyte/monocyte ratio; WBC, white blood count. Statistically significant p values are expressed in bold in the table.

Laboratory parameters required for index calculations were evaluated in the first trimester and compared between groups. Hemoglobin counts were significantly lower, whereas creatinine and monocyte counts were significantly higher in the preeclampsia group, with p-values of 0.005, 0.035, and 0.004, respectively. Neutrophil, lymphocyte, WBC, platelet, AST, and ALT counts were obtained similarly between the groups (Table 1).

When the indices were compared between the groups, SIRI, SII, APRI, LMR, NLR, PLR, and creatinine/platelet ratios were similar. The only statistically different index was the platelet/hemoglobin count ratio, with a p-value of 0.025 (Table 1).

Then, the preeclampsia group was further divided into subgroups according to the timing of diagnosis such as early-occurring and late-occurring preeclampsia. Eighty-nine early-occurring preeclampsia patients were diagnosed before 32 gestational weeks and 215 late-occurring preeclampsia patients. None of the determined indices was observed to be significantly different between groups in the first trimester (Table 2).

**Table 2:** Index comparison between early and late preeclampsia subgroups and between mild and severe preeclampsia subgroups.

Indices	Early preeclampsia (n=89)	Late preeclampsia (n=215)	pa-value	Mild preeclampsia (n=182)	Severe preeclampsia (n=122)	pb-value
SIRI	1.52 (1.13)	1.46 (0.98)	0.647	1.46 (1.0)	1.50 (1.0)	0.573
SII	877.94 (664.9)	799.44 (429.7)	0.432	807.29 (472.8)	821.09 (529.8)	0.692
APRI	0.063 (0.04)	0.060 (0.03)	0.939	0.059 (0.03)	0.063 (0.03)	0.347
NLR	3.00 (1.86)	3.00 (1.45)	0.774	3.07 (1.47)	2.96 (1.74)	0.909
PLR	144.31 (74.62)	134.13 (60.51)	0.546	133.03 (61.06)	144.83 (68.03)	0.163
LMR	4.16 (2.04)	4.00 (1.73)	0.680	4.25 (2.04)	3.94 (1.87)	0.089
Creatinine/platelet	0.002 (0)	0.002 (0)	0.425	0.002 (0)	0.002 (0)	0.115
Platelet/hemoglobin	21.56 (9.98)	21.49 (7.07)	0.326	21.56 (7.96)	21.45 (7.18)	0.957

All variables were presented as medians and interquartile ranges (IQR).

pa -value indicates comparisons between early and late preeclampsia groups, and

pb -value indicates comparisons between comparisons between mild and severe preeclampsia groups. SIRI, systemic inflammation response index; SII, systemic immune-inflammation index; APRI, AST to platelet ratio index; NLR, neutrophil/lymphocyte ratio; PLR, platelet/lymphocyte ratio; LMR, lymphocyte/monocyte ratio.

The preeclampsia subgroups included 122 severe and 182 mild preeclampsia patients. The indices were evaluated for severity prediction, and none of them was found to be significantly different. p-values are shown in Table 2.

**Figure 1:**
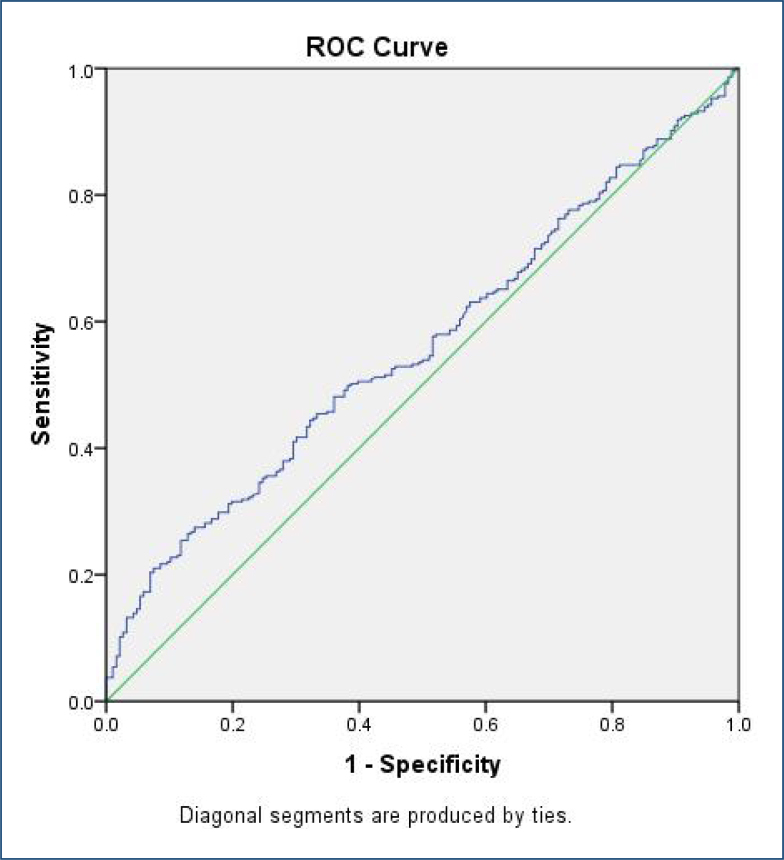
Receiver operating characteristic curve of platelet/hemoglobin count ratio on predicting preeclampsia development in the first trimester. The area under the curve was 0.561 (95% confidence interval=0.509–0.612, p=0.025). The optimal cut-off value of the platelet/hemoglobin count ratio was 21.41 (sensitivity=50%, specificity=60%).

ROC analyses were performed to determine optimal platelet/hemoglobin count ratio cut-off values. The optimal cut-off value of 21.41 was found to predict preeclampsia in the first trimester (Figure 1).

## DISCUSSION

In the present study, increased platelet, creatinine, and decreased hemoglobin counts were obtained in the preeclampsia group. The platelet/hemoglobin ratio was significantly higher in the preeclampsia group in the first trimester. SIRI or none of the other commonly used inflammation indices were found to be similar between groups. The platelet/hemoglobin ratio or other indices were found to be insufficient in predicting discriminating cases, according to the severity or occurring time in the evaluation of the first trimester.

Current findings indicated that preeclampsia was a vascular endothelial multisystem disorder, accompanied by platelet-induced microvascular thrombosis, capillary permeability increase, and hypertension^12^. The underlying disease etiology was suggested as placental hypoxia, leading to erythropoietic stimulation of bone marrow. Increased activated platelets and neutrophils accumulate in the intervillous space, stimulating endothelial dysfunction^13^. Increased inflammation and altered immune response act as etiological cofactors^14^.

Preeclampsia risk identification before clinical disease occurrence is an essential task due to its major complications, leading to fetal-maternal morbidity and mortality. Many studies about the inflammatory etiology of preeclampsia suggest that the inflammatory process occurs in early pregnancy, much before the clinical diagnosis of the disease. In the literature, an increased inflammatory process in the decidua was demonstrated in the first trimester of preeclampsia. Altered platelet reactivity was reported as an early marker for preeclampsia^15,16^.

In light of the first-trimester inflammation findings, studies focused on preeclampsia prediction and discrimination of high-risk pregnant women as early as possible. Commonly used blood parameters were evaluated and suggested as low-grade inflammation indicators. The PLR, LMR, NLR, APRI, and SII indices were frequently reported as valuable markers in predicting and diagnosing many diseases with inflammatory etiologies, including preeclampsia.

In the literature, conflicting results were reported on PLR. Some studies reported higher platelet and neutrophil counts and increased PLR in the preeclampsia group^2,10,14^. In the present study, the PLR between the groups did not differ, in discordance from the studies performed with smaller groups of patients in the literature. The platelet counts and lymphocyte counts were comparable between groups in concordance with recent studies that observed similar PLR counts in the preeclampsia group^17,18^.

LMR was found to be an indicator of subclinical inflammation and a prognostic marker for several diseases. In a study conducted on patients diagnosed with preeclampsia, predelivery LMR values were found to be significantly higher in the preeclampsia group^19,20^. The only study with a restricted patient number on LMR in the first trimester reported significantly lower LMR values for the preeclampsia group^21^. In this study, which was conducted as a complement to the study in the literature with a larger number of patients, monocyte counts were found to be lower. However, LMR values did not differ between the groups.

In most studies, NLR values were found to be elevated in the preeclampsia group, while in some studies, these values were similar^14,22^. In a small number of studies with a limited number of patients, NLR was found to be significantly different^23^. The presented study found that NLR values of the control and preeclampsia groups were similar.

The APRI score was reported as a better marker than AST alone for predicting HELLP syndrome^24^. APRI in preeclampsia was explored and found to be significant in the prediction of preeclampsia under 20 weeks of gestation^25^. We obtained comparable AST and ALT counts, like platelet counts, between groups. The APRI scores of the groups and subgroups were also similar. In our opinion, these parameter differences might occur later than the first trimester.

SII and SIRI indices may be considered relatively new markers that have been investigated in various diseases with an inflammatory etiology. These markers have been reported to be more efficient for predicting low-grade inflammation than traditional markers. In a recent study on 63 pregnant women diagnosed with preeclampsia, an elevated SII score was reported in the first-trimester evaluation^21^. To the best of our knowledge, this study was the first to explore SIRI in the prediction of preeclampsia. No significant difference was observed in SII or SIRI between groups or subgroups.

In the present study, creatinine counts were significantly higher in the preeclampsia group. Although the creatinine/platelet ratio was not found different between groups, increased creatinine might be an early indicator factor for preeclampsia's occurrence and an indicator of renal sensitivity of preeclampsia.

Lower hemoglobin counts were observed in the preeclampsia group, and this result was concordant with other studies in the literature^10^. Lower hemoglobin might be an early causative factor for the occurrence of preeclampsia. The platelet-to-hemoglobin ratio might be a useful index in the prediction of preeclampsia. In our study, this new ratio was significantly higher in the preeclampsia group compared to the control group.

This study was the first to explore SIRI, creatinine/platelet ratio, and platelet/hemoglobin ratio with the other commonly used inflammatory markers in the prediction of preeclampsia in the first trimester. One of the major strengths of this study was the evaluation time of blood parameters in the first trimester to predict subsequent preeclampsia. The relatively large sample size further strengthened the study. The possible limitation of this study was its retrospective design.

## CONCLUSION

Preeclampsia is a pregnancy-specific multisystem disorder that might lead to maternal-fetal morbidities and mortalities. Therefore, recent studies focused on predicting disease as early as possible. In this innovative study, the hypothesis was that the underlying inflammatory etiology of preeclampsia might begin to occur in the first trimester. The aim of this study was to evaluate possible inflammatory markers for prediction and to find the most appropriate marker or index. To the best of our knowledge, this was the first study to explore SIRI, creatinine to platelet ratio, and platelet to hemoglobin ratio with other inflammatory indices for the prediction of preeclampsia. The platelet/hemoglobin ratio was found to be a promising index predicting preeclampsia in the first trimester;,however, further studies are needed to confirm these findings.
